# Comparative Analysis of Volatile Aroma Compounds, Fatty Acids, and LOX Pathway Gene Expression of Two *Lentinula edodes* Mycelia

**DOI:** 10.3390/jof11120845

**Published:** 2025-11-28

**Authors:** Changxia Yu, Jun Jiang, Mengke Zhang, Qin Dong, Lin Yang, Lei Zha, Qian Guo, Yan Zhao

**Affiliations:** 1 National Engineering Research Center of Edible Fungi, Key Laboratory of Agricultural Genetics and Breeding of Shanghai, Institute of Edible Fungi, Shanghai Academy of Agricultural Sciences, Shanghai 201403, China; yuchangxia@saas.sh.cn (C.Y.); zmk1845342941@163.com (M.Z.); maomao88719@163.com (Q.D.); ylin_jade@163.com (L.Y.); zhalei@saas.sh.cn (L.Z.); guoqian@saas.sh.cn (Q.G.); 2 Lishui Institute of Agriculture and Forestry Sciences, Lishui 323000, China; jiangj-1@163.com

**Keywords:** *Lentinula edodes*, mycelia, aroma difference, lipoxygenase (LOX) pathway, mechanism elucidation

## Abstract

Although the aroma profile of *Lentinula edodes* has been extensively studied in fruiting bodies, the mycelial stage provides a distinct context for elucidating the fundamental metabolic pathways, free from the complexities of organismal development. To elucidate the mechanism underlying aroma differences between *L. edodes* strain 808 (the control strain) and its mutant strain ww808 (with almost no shiitake aroma), this study employed GC-IMS combined with PCA and OPLS-DA to identify key aroma biomarkers during the mycelial stage. All analyses were performed with three biological replicates. Furthermore, fatty acids composition, key enzyme activities of the LOX pathway, and their gene expression levels were systematically compared. The results indicated significant differences in the content of volatile aroma compounds in the mycelia of the two strains, primarily stemming from fundamental restructuring of gene expression and enzyme activity in the LOX pathway. The *LOX* gene expression and LOX activity of 808 mycelium were relatively high, facilitating the accumulation of key aroma compounds such as phenylethanal, benzaldehyde, and ethyl acetate, which constitute its distinctive aromatic profile. However, although the mycelium of ww808 possessed richer fatty acid precursor (C18:2), its lower *LOX* gene expression restricted the flux of this pathway. The significantly increased expression of *ADH2*, *ADH3*, and *ADH5* genes and higher ADH activity enhanced the conversion capacity of aldehydes to alcohols and ketones. Given the generally higher odor thresholds of alcohols and ketones compared to aldehydes, distinct aroma profiles emerged between the two strains. Pearson correlation analysis further confirmed the significant correlations between the aroma biomarkers, fatty acids, key genes, and enzyme activities. This study revealed the formation mechanism of aroma differences in the mycelia of the two strains from the perspective of metabolic pathways, providing a theoretical foundation and candidate targets for the directed genetic improvement of *L. edodes* aroma quality.

## 1. Introduction

*Lentinula edodes* (Berk.) Pegler, commonly known as shiitake mushroom, is one of the most widely cultivated and consumed edible fungi globally, highly regarded for its distinctive aroma, rich nutritional value, and health benefits [1]. Among the various factors determining its market value and consumer preferences, a intense and distinctive aroma often plays a more significant role than appearance, serving as a key evaluation criterion [2,3]. In China, the strain 808 is one of the most extensively cultivated and commercially dominant *L. edodes* varieties [4], celebrated by both producers and consumers for its robust growth, high yield, and intense aroma. Interestingly, a spontaneous mutant derived from 808, named ww808, exhibits a dramatic reduction in aroma quality despite maintaining similar morphological and growth characteristics.

The characteristic aroma of *L. edodes* is primarily derived from volatile organic compounds (VOCs), including sulfur-containing compounds, eight-carbon (C_8_) compounds, and various aldehydes, ketones, and alcohols [5,6,7]. The complex combination of these VOCs contributes its distinctive aroma profile [8]. The significant differences in aroma between strains 808 and ww808 suggest fundamental divergence in their volatile metabolism. Consequently, this pair of isogenic strains, which exhibits similar phenotypes but differing significantly in aroma production, provides a unique and ideal model system for investigating the molecular and biochemical mechanisms underlying aroma formation in *L. edodes*.

The biosynthetic pathways of aromatic compounds have been partially elucidated, with the lipoxygenase (LOX) pathway playing a crucial role in the formation of key aroma compounds [9,10,11]. This pathway begins with the oxidation of unsaturated fatty acids (UFAs), such as linoleic and linolenic acid, catalyzed by lipoxygenase (LOX). The resulting products are subsequently cleaved by hydroperoxide lyase (HPL) and further catalyzed by alcohol dehydrogenase (ADH), ultimately yielding volatile aldehydes and alcohols [12,13]. Accumulating evidence indicated that the expression levels of genes involved in the LOX pathway are closely associated with the synthesis of VOCs. For instance, changes in the expression of *PpLOX1*, *PpLOX3*, and *PpAAT1* had been linked to the loss of characteristic aroma in peach fruit under low temperature stress [14]. In tomatoes, the *ADH2* gene had been demonstrated to regulate the synthesis of aldehydic and alcoholic volatile flavor compounds [15]. In addition, we found that the VOCs in the mycelia of the two *L. edodes* strains were primarily aldehydes, C_8_ compounds, and alcohols. Based on this, we speculated that the activity of the LOX pathway and the composition of the precursor fatty acids pool are key factors determining the overall aroma intensity and profile of *L. edodes*.

In the analysis of VOCs, gas chromatography-ion mobility spectrometry (GC-IMS) has emerged as an ideal tool for comparing aroma fingerprints, owing to its high sensitivity, excellent reproducibility, and the ability to detect trace volatiles without complex pretreatment [16,17,18]. While previous studies have analyzed the VOCs during the fruiting stage of *L. edodes*, research focusing on the mycelial stage remains limited. The mycelial stage offers an advantage by eliminating confounding effects from developmental processes and environmental factors, thereby providing clearer insights into the intrinsic metabolic capabilities of a given strain.

In this study, GC-IMS was employed to compare the VOCs profiles of the 808 and ww808 during the mycelia stage, aiming to identify key differences in their aromatic compounds. Simultaneously, we analyzed the fatty acids compositions of two strains. Furthermore, we measured the activity of key enzymes in LOX pathway and analyzed the expression levels of related genes to systematically investigate the potential regulatory mechanisms underlying the observed differences in aroma. It should be noted that due to the complex intermediate products and rapid reaction of HPL catalysis, the specific substrates and forms of existence remain unclear [19], so we did not delve into the properties of HPL. Therefore, by integrating data from VOCs profiling, fatty acids compositions, enzyme activities, and genes expressions, this work attempts to elucidate the main mechanisms responsible for the aroma deficiency in the mutant ww808. The findings revealed the regulatory role of the LOX pathway in the formation of aroma differences between strains from an integrated metabolic pathway perspective for the first time, providing valuable insights into the regulation of aroma biosynthesis in *L. edodes*. The results not only provide new evidence for understanding the molecular basis of aroma formation in edible fungi, but also establish a theoretical foundation and potential targets for the genetic improvement of high-aroma varieties.

## 2. Materials and Methods

### 2.1. Materials and Mycelium Culture

The *L. edodes* strains 808 and ww808 were preserved at the Institute of Edible Fungi, Shanghai Academy of Agricultural Sciences. The strain ww808 was isolated from the 808 strain, and the fruiting bodies with almost no shiitake aroma appeared after 808 was cultivated. The strain was obtained through tissue isolation and named ww808. After isolation, the fruiting experiments of 808 and ww808 were conducted again for verification, and the results showed that the fruiting bodies of the two strains were only different in aroma, with no noticeable differences in appearance. Additionally, they could not be distinguished using SSR (Simple sequence repeats) molecular marker identification.

The mycelia were subcultured on a PDA (Potato dextrose agar) (Difco, Detroit, MI, USA) plate. After incubation for 10 d at 25 °C, the culture was transferred to 250 mL flasks containing 120 mL of PDB (Potato dextrose broth) (Difco, Detroit, MI, USA) medium. The mycelia were cultured in PDB medium and cultured at 25 °C with 150 rpm for 15 d. The mycelia were collected, washed with ddH_2_O, blotted with filter paper to remove excess moisture, and stored at −80 °C for later analysis. The samples of strains 808 and ww808 during the mycelial stage are represented by M808 and Mww808, respectively.

### 2.2. Volatile Compounds Analysis

Samples were analyzed in triplicate using a GC-IMS system (G.A.S. GmbH, Dortmund, Germany), configured with a WAX capillary column (15 m × 0.53 mm × 1 μm) (RESTEK, Bellefonte, PA, USA). The GC and IMS operational parameters were performed from the method of Xiang et al. with modifications [20]. 1 g sample of *L. edodes* was placed in a 20 mL headspace vial and incubated at 60 °C for 15 min, after which 500 μL of the headspace was injected into the injector using a heated syringe (85 °C) in splitless mode. GC conditions: Column temperature was 60 °C. Nitrogen gas (purity ≥ 99.999%) served as the carrier gas, at an initial gas flow rate of 2.0 mL/min. This speed was maintained for 2 min. Then, the speed was increased to 10.0 mL/min for 8 min and then to 100.0 mL/min for 10 min. The flow rate was maintained at this level for 40 min. Regarding the IMS conditions, a tritium source (^3^H) served as the ionizing source; the drift tube length was 53 mm; the electric field strength was 500 V/cm; the drift tube temperature was 45 °C; nitrogen gas (purity ≥ 99.999%) was the drift gas; and the drift gas flow rate was 75.0 mL/min. Detection was performed in positive ion mode. Both analyses were performed with three biological replicates. The identification of VOCs was performed by comparing both the GC retention time (calibrated against an n-ketone series to provide Retention Index, RI, values) and the IMS drift time with the reference standards in the built-in NIST/IMS database using the VOCal software (version 0.4.03) (G.A.S. GmbH, Dortmund, Germany). This two-dimensional matching provides a high degree of confidence in the identifications. A compound was considered positively identified when the library similarity score exceeded 80% and the experimental RI was within ± 15 units of the library RI value. It should be noted that these identifications, while based on a robust two-dimensional matching process, were not confirmed by injection of pure authentic standards for all compounds. Relative quantification was performed based on normalized peak areas.

### 2.3. Fatty Acids Analysis

Sample analysis was performed according to the method of Yu *et al*. [21], using an Agilent 7890A-5975C gas chromatography-mass spectrometry (GC-MS) instrument (Agilent Technologies, Santa Clara, CA, USA). Samples that had been stored at −80 °C were freeze-dried under vacuum conditions and then ground. Then, 0.1 g of each sample was added to a screw-cap ampere tube with 1.0 mL of 5% H_2_SO_4_ and 10 μL of the internal standard and mixed until homogeneous. Nitrogen gas (N_2_) was blown into the tube every 10 s to remove air. Then, the tube was immediately heated to 80 °C for 90 min. After cooling at room temperature, 1.5 mL of 0.9% NaCl and 400 μL of hexane were added, and after mixing thoroughly, the mixture was transferred to a centrifuge tube for centrifugation at 1000× *g* for 5 min. After static layering, 200 μL of the supernatant was transferred into an injection bottle. Both analyses were performed with three biological replicates.

The GC-MS analysis conditions were as follows. A DB-5ms chromatograpic column (30 m × 0.25 mm × 0.25 μm) was used. The injection volume was 1.00 μL, the injection temperature was 270 °C, the split ratio was 1:5, the carrier gas was helium (99.999%), and the flow rate was 1.0 mL/min. The column temperature program was as follows: the temperature was held at 90 °C for 5 min, increased at a rate of 8 °C/min until a temperature of 170 °C was reached, increased at a rate of 25 °C/min until a temperature of 290 °C was reached, and maintained at this value for 7 min. The interface temperature was 280 °C, the ion source temperature was 250 °C, and the quadrupole temperature was 150 °C. The ionization mode was electron impact (EI) at an energy level of 70 eV. The detector voltage was 2106 V. Full scan mode was used in the mass range of 33–500 *m*/*z*. The solvent delay was 3.2 min.

### 2.4. Analysis of Main Enzyme Activities in the LOX Pathway

LOX and ADH enzyme activity were performed according to the manufacturer’s instructions (Suzhou Mengxi Biomedical Technology Co., Ltd., Suzhou, China). All analyses were performed with four biological replicates.

### 2.5. Analysis of Gene Expression

The total RNA of the treated samples were extracted using a VeZol-Pure Total RNA Isolation Kit (VeZol-Pure Biotech Co., Ltd., Nanjing, China) according to the manufacturer’s instructions. And cDNAs were synthesized using PrimeScript FAST RT reagent kit with gDNA Eraser (TakaRa, Beijing, China). The quality and concentration of the extracted total RNA were assessed using a NanoDrop™ One/OneC Microvolume UV-Vis Spectrophotometer (Thermo Fisher Scientific, Waltham, MA, USA). Only RNA samples with an A260/A280 ratio between 1.8 and 2.2 and an A260/A230 ratio greater than 2.0 were used for subsequent cDNA synthesis. The integrity of the synthesized cDNA was verified by the successful and specific amplification of the reference gene in the qPCR assays.

The primers for *LOX* and *ADH*s were designed by Shanghai Sangong Biotech Co., Ltd. (Shanghai, China). The Beta-tubulin (*TUB*) was selected as the reference gene for normalization. Its selection was based on its verified stable expression in *L. edodes* mycelia under a range of experimental conditions, as established in our previous work [22]. Primers were synthesized by Tsingke Biotechnology Co., Ltd. (Beijing, China). The sequences are shown in Table S1.

RT-qPCR analyses were conducted according to the methods of Yang et al. [23]. The reactions utilized SYBR^®^ Premix Ex Taq^TM^ II (TaKaRa Biomedical Technology, Dalian, China) kit and were run on a StepOnePlus™ Real-Time PCR instrument (Applied Biosystems, Foster City, CA, USA). The reaction system was performed on ice, including 10 μL of SYBR^®^ Premix Ex Taq™ II (2×), 2 μL of template cDNA, 0.4 μL of ROX dye, 0.4 μL of each primer, and 6.8 μL of RNase-free water. The qRT-PCR amplification procedures were as follows: denaturation at 95 °C for 30 s, followed by 40 cycles of PCR at 95 °C for 5 s and 60 °C for 34 s; and a melting curve at 95 °C for 15 s, 60 °C for 1 min, and 95 °C for 15 s. Each sample was analyzed with three biological replicates. The relative expression levels of target genes were quantified based on the 2^−∆∆Ct^ method.

### 2.6. Statistical Analysis

Analysis of variance (ANOVA) was performed with SPSS 19.0, and multiple comparisons were performed with Duncan’s test.

## 3. Results

### 3.1. GC-IMS Analysis of VOCs in Two L. edodes Mycelia

**Figure 1:**
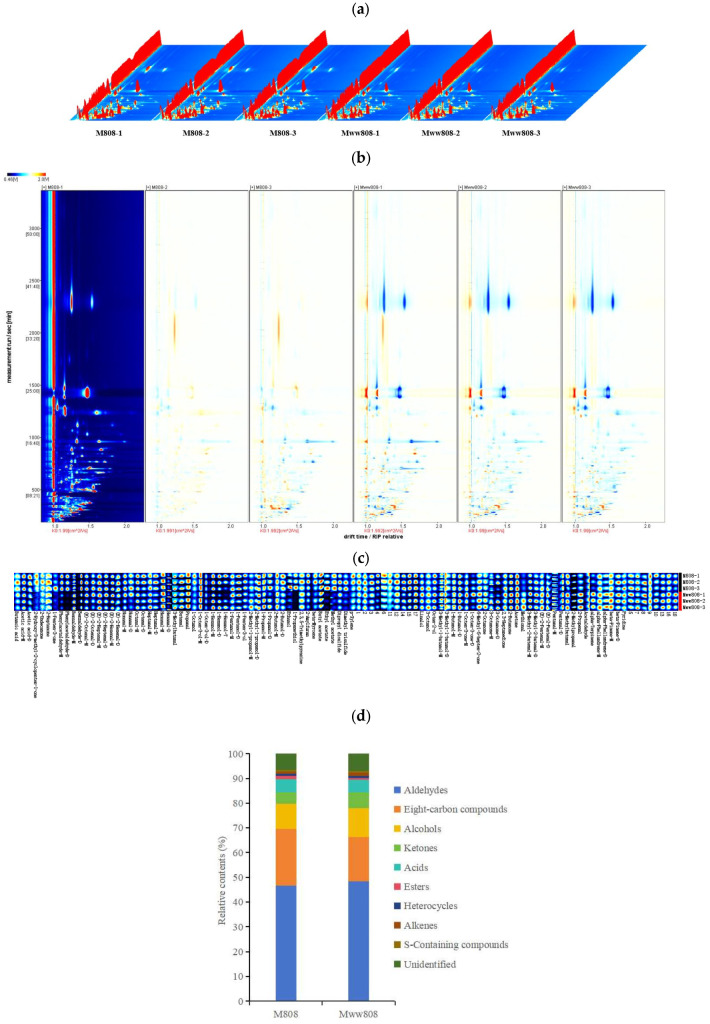
VOCs in mycelia of *L. edodes* by GC-IMS. (**a**) Three-dimensional GC-IMS images; (**b**) two-dimensional GC-IMS difference plot; (**c**) gallery plot fingerprint (**d**) relative contents (%) of volatile organic compounds. The number ‘1’, ‘2’, and ‘3’ appended to each sample identifier indicate the serial numbers of these individual replicates. The colors in (**a**) represented the concentration of compounds, with white indicating lower concentration, red indicating higher concentration, and the darker the color indicating the higher concentration. Blue and red in (**c**) indicated that the concentration of the compound was lower and higher than the reference, respectively, and the darker the color represented the greater difference.

GC-IMS technology was used to analyze the characteristic VOCs of two *L*. *edodes* strains in mycelial stage. The three-dimensional IMS spectra provided an overview of VOCs profile in the two strains. As shown in Figure 1a, the number of detected VOCs was generally similar between the two strains, but their relative contents differed. The difference profiles in VOCs between the two strains were further compared intuitively through the VOCs difference spectrum (Figure 1b). Using M808-1 as the reference, the difference spectrum was obtained by subtracting its spectral signal peak. It can be seen that the types of VOCs between the two strains were generally consistent, but there were significant differences in concentrations.

The GC-IMS equipped with the Gallery Plot plugin was used to further compare the differences in the content of VOCs between the two *L*. *edodes* mycelia. Fingerprint was plotted based on the identified content and composition of VOCs (Figure 1c). The differences in VOCs between the two mycelia can be intuitively observed from the fingerprint. As shown in Figure 1c, it was consistent with the peak area results, the VOCs contained in the two mycelia were quite similar, but there was a significant difference in the concentrations of VOCs between the strains. These compounds can be used to distinguish the differences between the two *L*. *edodes* mycelia.

VOCal software was used to analyze the spectral data, and qualitative and quantitative analysis were performed. Compounds were identified using the built-in NIST and IMS software databases. A total of 105 VOCs were detected in the two strains, including 23 aldehydes, 19 alcohols, 18 eight carbon (C_8_) compounds, 7 ketones, 7 hydrocarbons, 4 sulfur-containing compounds, 3 esters, 3 heterocyclic compounds, 3 acids, and 18 unknown compounds. The VOCs were classified, and the peak volumes of each category were calculated to draw the relative content distribution of two mycelia. As shown in Figure 1d, there was a significant difference in the content of VOCs between the two mycelia, with aldehydes accounting for the highest proportion, followed by C_8_ compounds and alcohols. These compounds constituted 46.54%, 22.98%, 10.21% of the VOCs in 808 and 48.29%, 18.06%, 11.74% of the VOCs in ww808, respectively, totaling 79.73% and 78.09%, indicating they were the main VOCs in the two *L. edodes* mycelia.

Normalizing the peak areas of all VOCs in the fingerprint to obtain the relative contents, the differences in VOCs between the two mycelia were clearly presented (Supplementary Table S2). The relative content of C_8_ compounds in the 808 was significantly higher than that in ww808, while the proportions of aldehydes and alcohols were lower in 808 than those in ww808. This might be the underlying reason for the differences in aroma between the two strains. Aldehydes detected with relatively high content in both mycelia included benzaldehyde, hexanal, pentanal, 2-methylbutanal, and (E)-2-heptenal. C_8_ compounds detected with relatively high content in both mycelia included phenylacetaldehyde and 1-octen-3-ol. Phenylacetaldehyde has the sweetness of rose and honey [24], while 1-octen-3-ol is commonly known as mushroom alcohol, which exhibits a mushroom-like odor and is an important aroma compound in fresh shiitake. The highest relative content of alcohols detected in both mycelia was 1-hexanol, which has a sweet and oily aroma.

### 3.2. Screening and Identification of Potential Aroma Biomarkers in Two L. edodes Mycelia

**Figure 2:**
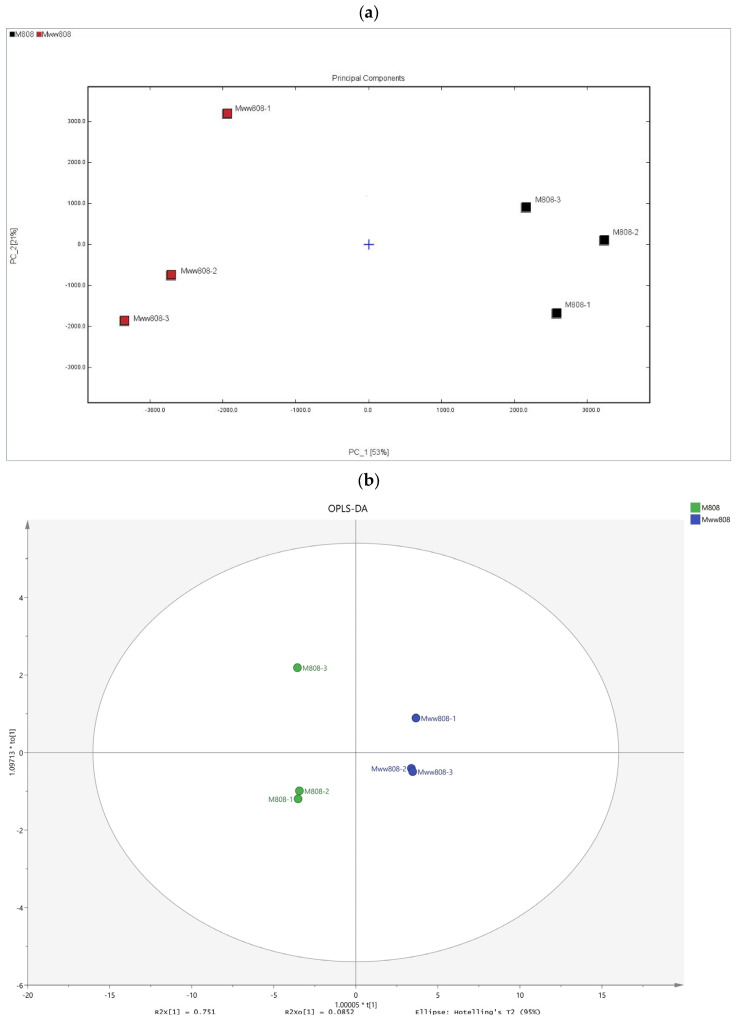

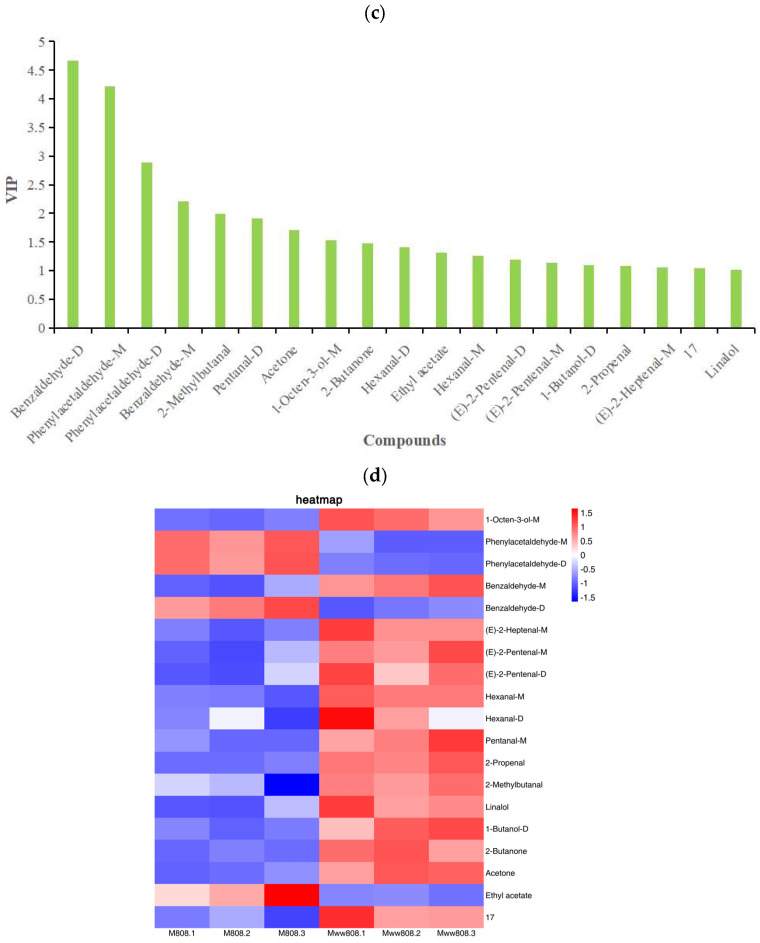
Similarity analysis and OPLS-DA of volatile organic compounds in mycelia of 808 and ww808. (**a**) PCA score diagram; (**b**) OPLS-DA score chart of the volatile organic compounds; (**c**) predicted value of VIP; (**d**) cluster heatmap.

In this study, Principal Component Analysis (PCA) was performed on the VOCs of the two *L. edodes* mycelia using the peak intensity values corresponding to the characteristic peaks as parameter variables. As shown in Figure 2a, the contribution rate of PC1 was 53%, and that of PC2 was 21%, with a cumulative contribution rate reaching 74%, which can effectively reflect the overall information. The close distances between sample replicates indicated there was a good reproducibility of the sample detection. The two groups of samples were distributed independently on the PCA distribution map, suggesting significant differences between the two strains.

OPLS-DA is a regression modeling method based on orthogonal partial least squares discriminant analysis. After visualization analysis of the 105 VOCs from the two mycelia, an OPLS-DA model was constructed to screen for VOCs with VIP values greater than 1 as differential VOCs between the two strains. The overall adaptability and predictive ability of the OPLS-DA model were evaluated using *R*^2^*X* and *Q*^2^ values. In this study, the cumulative *R*^2^*X*, *R*^2^*Y*, and *Q*^2^ values were 0.836, 0.999, and 0.993, respectively (Figure 2b), indicating that the established OPLS-DA model had good predictive ability (*Q*^2^ > 0.50). It can distinguish the mycelia of 808 and ww808 based on the detected VOCs and clarify the differences between them. Considering the possibility of overfitting in OPLS-DA, 200 permutation tests were conducted to validate the reliability of model.

Based on the OPLS-DA model, the VIP values of VOCs were calculated, and characteristic differential markers were selected according to the VIP values. After analysis and screening, a total of 19 VOCs with VIP values greater than 1 were found in two mycelia (Figure 2c), indicating that these compounds contribute significantly to the aroma of the two strains. These volatile compounds were mainly aldehydes and C_8_ compounds, among which aldehydes (VIP value of 17.90) included: benzaldehyde-D (4.67), benzaldehyde-M (2.20), 2-methylbutanal (1.99), pentanal-D (1.91), hexanal-D (1.40), hexanal-M (1.26), (E)-2-pentenal-D (1.19), (E)-2-pentenal-M (1.14), acrolein (1.08), and (E)-2-heptenal-M (1.06); C_8_ compounds (VIP value of 8.63) included: phenylacetaldehyde M (4.21), phenylacetaldehyde D (2.89), and 1-octen-3-ol M (1.53). In order to visually reflect the differences in characteristic differential markers between the two mycelia, a heatmap was drawn based on the relative contents of 19 selected characteristic differential markers (Figure 2d). The results indicated that phenylacetaldehyde-M, phenylacetaldehyde-D, benzaldehyde-D, and ethyl acetate were present in higher contents in 808 mycelium, while the other 15 VOCs were higher in ww808 mycelium.

### 3.3. Fatty Acids Analysis in Two L. edodes Mycelia

**Figure 3:**
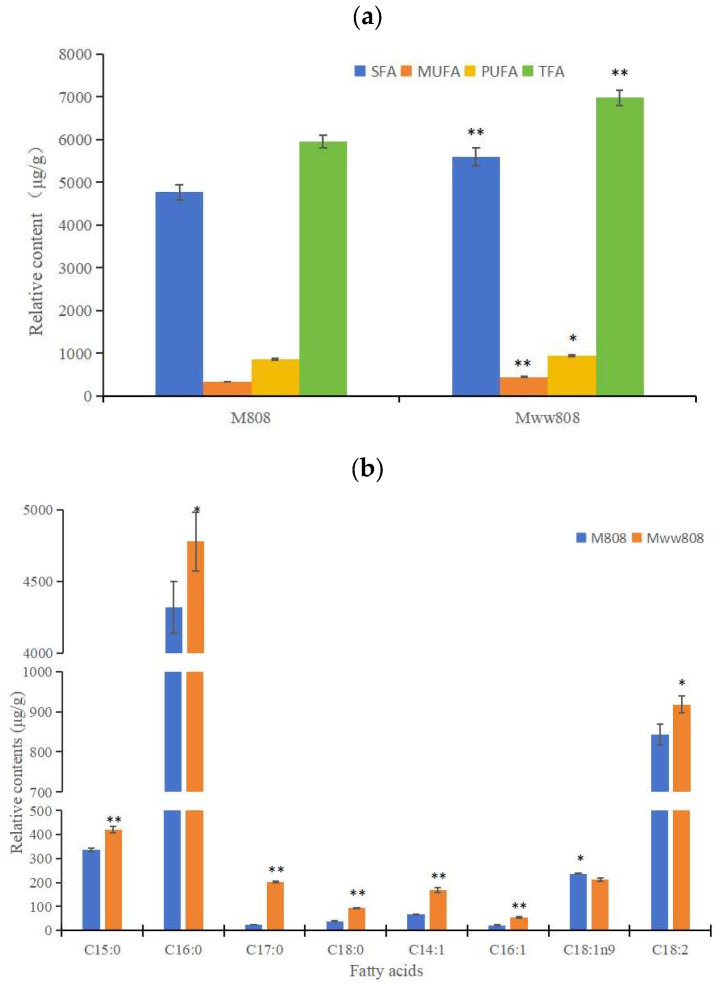
Relative contents of fatty acids in mycelia of 808 and ww808. (**a**) overall distribution of fatty acids; (**b**) relative contents of eight major fatty acids. * indicates significant correlation at the level of 0.05; ** indicates significant correlation at the level of 0.01.

The detection results of fatty acids in the 808 and ww808 mycelia were shown in Supplementary Table S3. A total of 34 fatty acids were identified in the two mycelia, including 17 saturated fatty acids (SFA), 8 monounsaturated fatty acids (MUFA), and 9 polyunsaturated fatty acids (PUFA). The fatty acids components were identical between the two mycelia, but the contents were different, with the most abundant of palmitic acid (C16:0) among all components. As shown in Figure 3a, the total fatty acids (TFA) content in 808 and ww808 mycelia was 5954.61 μg/g and 6977.76 μg/g, respectively. SFA were predominant, accounting for 80.05% and 80.16% of TFA content in the two strains, respectively. The contents of SFA, MUFA, PUFA, and TFA in the ww808 mycelia were all higher than those in 808.

The relative contents of eight fatty acids in the 808 and ww808 mycelia were high, accounting for 98.77% and 98.09% of TFA content of two mycelia, respectively (Figure 3b). They included four SFAs (pentadecanoic acid (C15:0), palmitic acid (C16:0), heptadecanoic acid (C17:0), stearic acid (C18:0)), three MUFAs (tetradecenoic acid (C14:1), palmitoleic acid (C16:1), oleic acid (C18:1n9)), and one PUFA (linoleic acid (C18:2)). Among the UFAs, C18:2 was the most abundant, accounting for 70.90% and 66.31% of the UFAs content in 808 and ww808 mycelia, respectively. The relative content of C18:1n9 was higher in the 808 mycelium, while the relative contents of the other seven fatty acids were higher in the ww808 mycelium.

### 3.4. Analysis of Gene Expression and Key Enzyme Activities Related to the LOX Pathway

**Figure 4:**
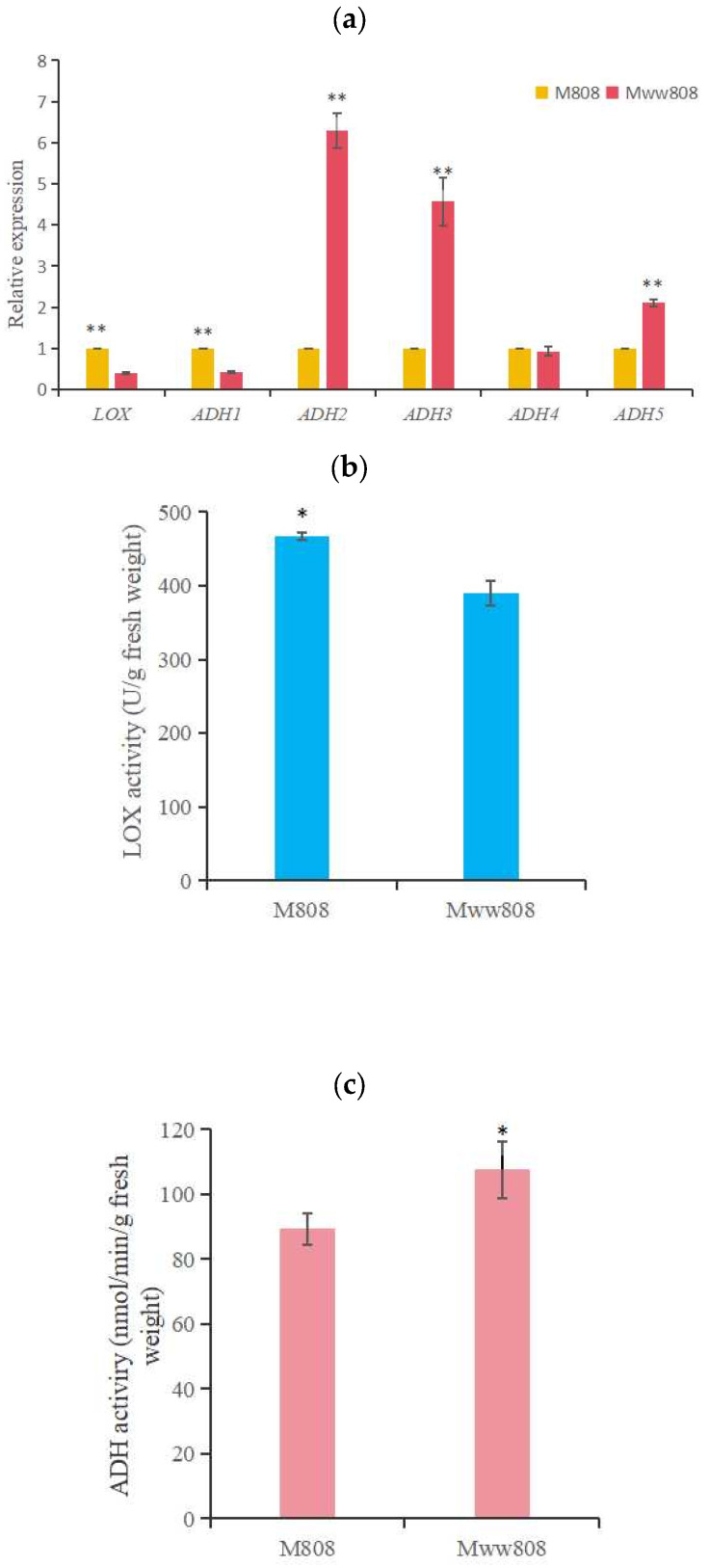
Gene expression of *LOX* and *ADHs*, and enzyme activities of LOX and ADH in mycelia of 808 and ww808. (**a**) gene expressions; (**b**) LOX activity; (**c**) ADH activity. * indicates significant correlation at the level of 0.05; ** indicates significant correlation at the level of 0.01.

Fatty acids metabolism is one of the main pathways for the production of VOCs. Most VOCs are generated through the oxidative degradation of fatty acids, which are regulated by various enzymes and genes, resulting in the formation of small molecular VOCs such as aldehydes and ketones [25]. We compared the expression levels of genes related to the LOX pathway (*LOX* and *ADHs*) and the activities of LOX and ADH enzymes in the two *L. edodes* mycelia. As shown in Figure 4a, there was a significant difference in the expression level of *LOX* between the two strains, and the expression level of 808 was higher than that of ww808. There were also significant differences in the expression levels of *ADH1*, *ADH2*, *ADH3*, and *ADH5* between the two strains. Specifically, the expression level of *ADH1* in 808 was higher than that in ww808, while the expression levels of *ADH2*, *ADH3*, and *ADH5* were lower than those in ww808. There was no significant difference in the expression level of *ADH4* between the two strains. There was a significant difference in LOX activity between 808 and ww808 (Figure 4b), and 808 was higher than ww808, which was consistent with the *LOX* expression levels of the two strains. The difference in ADH activity between 808 and ww808 mycelia was significant (Figure 4c), and 808 was lower than ww808, which was consistent with the expression levels of *ADH2*, *ADH3*, and *ADH5*.

### 3.5. Correlation Analysis Between Aroma Compounds and Fatty Acids, LOX Pathway Related Genes, and Enzyme Activities in Two L. edodes Mycelia

**Figure 5:**
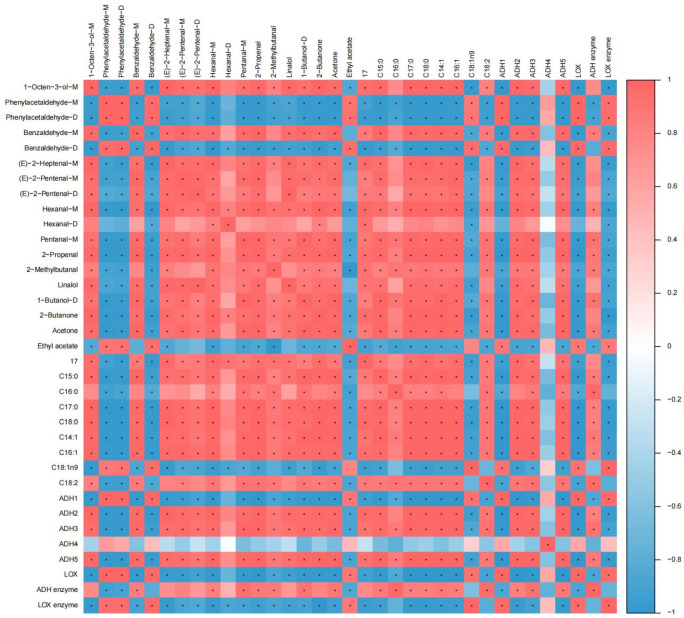
Pearson correlation analysis of aroma compounds in mycelia of 808 and ww808. * indicates significant correlation at the level of 0.05.

To further investigate the correlations between fatty acids, LOX pathway related genes and enzyme activities with the aroma markers, Pearson analysis was performed (Figure 5). Phenylacetaldehyde-M, phenylacetaldehyde-D, benzaldehyde-D, and ethyl acetate in mycelia were significantly positively correlated with C18:1 n9, *ADH1*, *LOX*, and LOX (*p* < 0.05), and significantly negatively correlated with the other fatty acids, *ADH2*, *ADH3*, *ADH5*, and ADH (*p* < 0.05). 1-Octen-3-ol-M, benzaldehyde-M, (E)-2-heptenal-M, (E)-2-pentenal-M, (E)-2-pentenal-D, hexanal-M, pentanal-M, acrolein, 2-methylbutanal, linalool, 1-butanol-D, 2-butanone, and acetone were significantly positively correlated with C15:0, C17:0, C18:0, C14:1, C16:1, C18:2, *ADH2*, *ADH3*, and *ADH5* (*p* < 0.05), and significantly negatively correlated with C18:1 n9, *ADH1*, *LOX*, and LOX (*p* < 0.05), among which benzaldehyde-M, hexanal-M, pentanal-M, acrolein, 2-methylbutanal, 1-butanol-D, and acetone were also significantly positively correlated with ADH enzyme activity (*p* < 0.05). These findings indicated that the eight major fatty acids, *ADHs*, *LOX*, ADH and LOX were strongly correlated with most of the volatile aroma markers in the two *L. edodes* mycelia.

## 4. Discussion

In this study, a comprehensive comparative analysis of the VOCs was conducted during the mycelial stage of two *L*. *edodes* strains, 808 and its mutant ww808. It is important to note that the metabolic differences and mechanisms discussed herein are specific to the mycelial stage of the fungi, analyzed after a standardized 15 d cultivation period in liquid medium. This specific time point was selected to capture the metabolically active vegetative growth stage, thereby allowing a direct comparison of the intrinsic metabolic capabilities of the two strains without the confounding influences of fruiting body development or senescence. By integrating data on VOCs, FAs, and the enzymatic activities and gene expression levels of the LOX pathway, we identified the key metabolites at the mycelial stage and elucidated the underlying causes of the aroma differences between 808 and ww808.

The most direct evidence came from GC-IMS analysis, which effectively established volatile fingerprints for both strains during mycelial stage. A total of 105 VOCs (including 18 unknown compounds) were detected, mainly including aldehydes, C_8_ compounds, and alcohols, with consistent VOCs types between the two strains. This finding aligns with previous studies indicating that aldehydes and C_8_ compounds are major contributors to the characteristic aroma of *L*. *edodes* [26,27,28]. Aldehydes mainly originated from the degradation of fatty acids, which are produced through oxidation and degradation of fatty acids. Due to their low odor thresholds [29], they usually play an important role in the aroma of edible mushrooms. C_8_ compounds are important aroma compounds in *L. edodes*, with a low odor thresholds [30]. They are mainly synthesized in edible fungi through the oxidation of free linoleic acid and catalytic cracking by enzymes [31,32]. The odor thresholds of alcohols were relatively high [33], and their synthesis mainly derived from the oxidation of lipids or the reduction of carbonyl compounds. However, there was a significant difference in the content of VOCs during the mycelial stages between the two strains, with 808 having a significantly higher relative content of C_8_ compounds and a lower relative content of aldehydes compared to ww808.

Using an OPLS-DA model, 19 VOCs with VIP scores greater than 1 were identified, mainly including benzaldehyde, phenylacetaldehyde, 2-methylbutanal, pentanal, 1-octen-3-ol, among others. These compounds were the key VOCs distinguishing the aroma differences between the two strains. The differences in their content and composition constitute the distinct aroma profiles of 808 and ww808 mycelia, respectively. Further correlation heatmap analysis revealed that phenylacetaldehyde-M, phenylacetaldehyde-D, benzaldehyde-D, and ethyl acetate were present in higher levels in 808 mycelia, while the other 15 VOCs were more abundant in ww808 mycelia. These results clearly revealed the material basis for the differences in volatile aroma characteristics between the two strains.

Based on the fact that the LOX pathway is the main synthesis pathway for many VOCs, particularly C_8_ compounds, we speculated that the aforementioned differences might stem from changes in fatty acid precursors or LOX pathway related genes expressions and enzyme activities. Fatty acids composition analysis showed that SFAs were predominant in the mycelia of both strains, which was inconsistent with our previous research results on other strains of *L*. *edodes*. The fatty acids measured in the mycelia of strains 18 and 18N44 were mainly UFAs [21], which might be related to the genetic characteristics and growth environment of the strains themselves. The content of SFAs, MUFAs, PUFAs, and TFAs in 808 mycelium was significantly lower than that in ww808 mycelium, with the relative content of eight fatty acids being higher in the two mycelia. It is worth noting that the relative content of the key UFAs, C18:1n9 was higher in 808 mycelium, whereas C18:2 (a key substrate in the LOX pathway) was more abundant in ww808 mycelium. This demonstrated that the ww808 mycelium had a greater reserve of precursors for synthesizing volatile aroma compounds.

Gene expression and enzyme activity assays provided key evidence for elucidating the underlying mechanism. On the one hand, LOX was a crucial rate limiting enzyme in the LOX pathway, LOX expression level and LOX activity in ww808 mycelium were significantly lower than those in 808 mycelium. This downregulation of *LOX* in ww808 limited its capacity to catalyze the generation of hydroperoxides from UFAs through the LOX pathway, which could be subsequently converted into C_8_ compounds and aldehydes. The result was consistent with the lower levels of key aroma compounds such as phenylacetaldehyde and benzaldehyde detected in ww808 mycelium. It is particularly noteworthy that although ww808 was richer in the LOX substrate linoleic acid (C18:2), the low LOX activity created a bottleneck in the synthesis of VOCs through the LOX pathway in ww808, which may be due to transcriptional regulation failure or post-translational modification abnormalities, resulting in its inability to effectively utilize substrate resources. On the other hand, ADH activity was significantly higher in ww808 mycelium, accompanied by significantly upregulated expression of ADH2, ADH3, and ADH5. This enhanced reductive capacity in mutant ww808 mycelium favored the conversion of available aldehydes into alcohols. However, in 808 mycelium, aldehydes and C_8_ compounds with low odor thresholds accumulated, constituting its distinctive aroma profile. Consequently, the pronounced conversion of aldehydes to alcohols in ww808, coupled with the generally higher odor thresholds of alcohols, resulting in a weakened overall aroma intensity and ultimately forming a aroma profile distinct from that of 808.

The strong correlations between major fatty acids, LOX pathway related genes and enzymes, and volatile aroma markers were further established by Pearson correlation analysis. Phenylacetaldehyde-M, phenylacetaldehyde-D, benzaldehyde-D, and ethyl acetate in the mycelia were significantly positively correlated with C18:1 n9, ADH1, LOX, and LOX (*p* < 0.05), indicating that their accumulation in 808 mycelium was mainly mediated by LOX and ADH1, and ADH activity was mainly driven by *ADH1*, but it was insufficient to convert a large amounts of aldehydes into alcohols. 1-Octen-3-ol-M, benzaldehyde-M, (E)-2-heptenal-M, (E)-2-pentenal-M, (E)-2-pentenal-D, hexanal-M, pentanal-M, acrolein, 2-methylbutanal, linalool, 1-butanol-D, 2-butanone, and acetone in the mycelia were significantly positively correlated with C15:0, C17:0, C18:0, C14:1, C16:1, C18:2, ADH2, ADH3, and ADH5 (*p* < 0.05). Among those, benzaldehyde-M, hexanal-M, pentanal-M, acrolein, 2-methylbutanal, 1-butanol-D, and acetone were also significantly positively correlated with ADH activity (*p* < 0.05), and were significantly negatively correlated with LOX expression and LOX activity (*p* < 0.05). This suggested that the formation of these compounds in ww808 might rely on alternative pathways independent of typical LOX pathway, potentially involving alternative oxylipin pathways or non-enzymatic oxidation processes. Under the influence of highly expressed ADH2, ADH3, ADH5 and elevated ADH enzyme activity, some aldehydes were further converted into alcohols and ketones, thereby achieving a dynamic equilibrium of aldehydes, alcohols, and ketones.

The metabolic perturbations observed in ww808, characterized by the accumulation of fatty acid precursors and an increase in alcohols, may extend their influence beyond the aroma profiles to broader fungal physiology. The accumulated UFAs, owing to the bottleneck in the LOX pathway, could serve as reservoirs for membrane lipid synthesis, potentially influencing cellular integrity and stress tolerance [34]. Concurrently, the elevated synthesis of alcohols, driven by high *ADH2*, *ADH3*, and *ADH5* expression, may play a role in maintaining cellular redox balance by regulating the NAD^+^/NADH ratio [35]. While the primary focus of this study is the elucidation of aroma divergence, these metabolic changes suggested a profound reprogramming of central metabolism in ww808, which could potentially impact its developmental fitness and environmental adaptation, warranting further investigation.

## 5. Conclusions

In this study, we systematically elucidated the mechanisms underlying the differences in volatile aroma profiles during the mycelial stage between the *L*. *edodes* strain 808 and its mutant strain ww808. Our findings demonstrated that the aroma differences mainly stem from fundamental variations in LOX pathway related gene expression and enzyme activities. Strain 808 exhibited high *LOX* gene expression and strong LOX enzyme activity, leading to substantial accumulation of key VOCs such as phenylacetaldehyde, benzaldehyde, and ethyl acetate, which underpinned its distinctive aroma profile. However, although ww808 had more abundant fatty acid precursors (C18:2), the low expression of its *LOX* gene created a bottleneck in the synthesis of C_8_ and other related compounds through the LOX pathway. Instead, ww808 may generate a variety of aldehydes through alternative pathways. The high expression of *ADH2*, *ADH3*, and *ADH5* genes, along with significantly increased ADH enzyme activity, facilitating a rapid conversion of aldehydes into abundant alcohols and ketones. This shift ultimately established a dynamic balance among aldehydes, alcohols, and ketones, resulting in an aroma profile distinct from strain 808. The odor thresholds of alcohols and ketones are generally much higher than those of aldehydes, resulting in specific differences in the content and composition ratio of aroma compounds between the two strains. This research not only clarifies the mechanistic basis for aroma formation differences between 808 and ww808 mycelia, but also provides a theoretical foundation for molecular genetic improvements in the aroma quality of edible fungi.

## Data Availability

Data will be made available on request.

## Supplementary Materials

The following supporting information can be downloaded at: https://www.mdpi.com/article/10.3390/jof11120845/s1, Table S1: Primers used for RT-qPCR; Table S2: Composition and content of volatile organic compounds in mycelia of *L. edodes* 808 and ww808; Table S3: Fatty acids composition during mycelial stage of *L. edodes* 808 and ww808.
