# Supplementary material for: Comparative Analysis of Volatile Aroma Compounds, Fatty Acids, and LOX Pathway Gene Expression of Two Lentinula edodes Mycelia
Source: J Fungi (Basel). 2025 Nov 28;11(12):845. doi: 10.3390/jof11120845 (PMC12733506; doi:10.3390/jof11120845)
Supplement: Supplementary file 1 [file jof-11-00845-s001.zip › jof-3964385-supplementary.pdf]

Table S1. Primers used for RT-qPCR

Gene	Forward（5′-3′）	Reverse:（5′-3′）
LOX	GGTCGCTATCCATCTCGTCAACAC	GTGCTCCAGTGAGGTTCCAACAG
ADH1	CTTGATTGGTGGCATTGCGGAAAC	CCTGAACTTGACCTGGGACTTGAC
ADH2	CAACTGCATCGTCCGTCCACTC	TCCTATGTTCCAACGCAGCCAATC
ADH3	CAGGTGCGGTTCATCCAGGTTC	TGGGTCGGAGATGGTGGTATTGG
ADH4	TTTACGGCATCGCTGGCTCAC	CGGAACGACGGACTCTTGTAGTTG
ADH5	TCCGTGCCTCCTGTCCAGTTG	GCGTAAGAGCCGTCGATACCAAG
TUB	GACATTTGCTTCCGAACCCT	CGGACATAACAAGGGACACA

Table S2. Composition and content of volatile organic compounds in mycelia of *L. edodes* 808 and ww808

**Category**	**Compound**	**CAS**	**RI**	**Comment**	**Molecule formula**	**Peak area**	
						**808**	**ww808**
S-Containing compounds	Dipropyl disulfide	C629196	1419.2		C6H14S2	0.35±0.03	0.36±0.05
	1-Propanethiol	C107039	849.4		C3H8S	0.13±0.02*	0.02±0.00
	Dimethyl trisulfide	C3658808	1340.0		C2H6S3	0.23±0.04*	0.12±0.01
	Methional	C3268493	1476.5		C4H8OS	0.10±0.00	0.26±0.04**
	Total					0.82±0.07	0.77±0.01
Eight-carbon compounds	1-Octanol	C111875	1661.1		C8H18O	0.19±0.01	0.21±0.01
	1-Octen-3-ol-M	C3391864	1487.8	Monomer	C8H16O	3.16±0.02	3.76±0.05**
	1-Octen-3-ol-D	C3391864	1488.5	Dimer	C8H16O	3.38±0.39	3.37±0.22
	(E)-2-Octenal-M	C2548870	1440.1	Monomer	C8H14O	1.30±0.06	1.42±0.16
	(E)-2-Octenal-D	C2548870	1440.8	Dimer	C8H14O	0.42±0.04	0.42±0.10
	3-Octanol	C589980	1448.0		C8H18O	0.14±0.01	0.19±0.01*
	Octan-2-ol	C123966	1340.8		C8H18O	0.40±0.03	0.52±0.00**
	Phenylacetaldehyde-M	C122781	1773.8	Monomer	C8H8O	8.36±0.37**	3.68±0.50
	Phenylacetaldehyde-D	C122781	1775.8	Dimer	C8H8O	2.67±0.20**	0.49±0.07
	1-Octen-3-one-M	C4312996	1310.1	Monomer	C8H14O	0.63±0.05	0.85±0.12**
	1-Octen-3-one-D	C4312996	1310.1	Dimer	C8H14O	0.22±0.05	0.36±0.15**
	Octanal-M	C124130	1298.3	Monomer	C8H16O	0.58±0.04	0.68±0.01*
	Octanal-D	C124130	1297.7	Dimer	C8H16O	0.19±0.03	0.18±0.00
	2-Octanone	C111137	1292.9		C8H16O	0.10±0.00	0.15±0.01**
	3-Octanone-M	C106683	1261.4	Monomer	C8H16O	0.53±0.08	0.77±0.14**
	3-Octanone-D	C106683	1259.8	Dimer	C8H16O	0.55±0.08	0.81±0.26**
	p-Xylene	C106423	1134.7		C8H10	0..10±0.01	0.10±0.01
	6-Methyl-5-hepten	C110930	1346.2		C8H14O	0.06±0.00	0.09±0.00**

	-2-one						
	Total					22.98±0.05**	18.06±0.93
Aldehydes	Benzaldehyde-M	C100527	1551.8	Monomer	C7H6O	5.39±0.17	6.68±0.11**
	Benzaldehyde-D	C100527	1552.8	Dimer	C7H6O	18.79±0.62**	13.11±0.37
	Nonanal-M	C124196	1403.0	Monomer	C9H18O	0.69±0.03	0.69±0.12
	Nonanal-D	C124196	1403.0	Dimer	C9H18O	0.11±0.01	0.09±0.04
	(E)-2-Heptenal-M	C18829555	1329.8	Monomer	C7H12O	1.21±0.02	1.50±0.04**
	(E)-2-Heptenal-D	C18829555	1331.0	Dimer	C7H12O	1.54±0.08	1.68±0.28
	(E)-2-Hexenal-M	C6728263	1224.2	Monomer	C6H10O	0.51±0.01	0.65±0.03*
	(E)-2-Hexenal-D	C6728263	1224.2	Dimer	C6H10O	0.72±0.02	0.70±0.02
	3-Methyl-2-butenal-M	C107868	1204.5	Monomer	C5H8O	0.18±0.00	0.28±0.01**
	3-Methyl-2-butenal-D	C107868	1205.9	Dimer	C5H8O	0.12±0.01	0.18±0.01**
	Heptanal-M	C111717	1181.2	Monomer	C7H14O	0.40±0.01	0.46±0.03
	Heptanal-D	C111717	1183.3	Dimer	C7H14O	0.39±0.03	0.37±0.01
	(E)-2-Pentenal-M	C1576870	1138.1	Monomer	C5H8O	0.60±0.06	0.95±0.04**
	(E)-2-Pentenal-D	C1576870	1138.1	Dimer	C5H8O	0.89±0.09	1.29±0.08*
	Hexanal-M	C66251	1097.8	Monomer	C6H12O	1.30±0.03	1.71±0.02**
	Hexanal-D	C66251	1099.1	Dimer	C6H12O	5.15±0.19	5.79±0.27
	Pentanal-M	C110623	987.1	Monomer	C5H10O	0.30±0.01	0.45±0.02**
	Pentanal-D	C110623	987.3	Dimer	C5H10O	2.38±0.07	3.33±0.02**
	Butanal	C123728	883.3		C4H8O	1.09±0.10	1.33±0.04*
	2-Propenal	C107028	856.6		C3H4O	0.27±0.01	0.58±0.02**
	Acetaldehyde	C75070	735.9		C2H4O	0.60±0.02	0.81±0.05**
	2-Methylbutanal	C96173	942.2		C5H10O	1.66±0.41	2.81±0.07*
	3-Methylbutanal	C590863	923.6		C5H10O	0.99±0.02	1.26±0.04**
	2-Methylpropanal	C78842	811.6		C4H8O	0.24±0.02	0.37±0.02**
	Propanal	C123386	798.9		C3H6O	1.01±0.05	1.23±0.06*
	Total					46.54±0.27	48.29±0.40**
Alcohols	Linalol	C78706	1645.9		C10H18O	0.53±0.05	0.81±0.04**
	1-Hexanol-M	C111273	1370.3	Monomer	C6H14O	1.57±0.11	1.81±0.35
	1-Hexanol-D	C111273	1371.1	Dimer	C6H14O	0.95±0.18	0.98±0.05
	1-Hexanol-T	C111273	1371.1	Trimer	C6H14O	0.22±0.07	0.21±0.09
	1-Pentanol-M	C71410	1263.1	Monomer	C5H12O	0.55±0.02	0.66±0.05*
	1-Pentanol-D	C71410	1262.3	Dimer	C5H12O	0.29±0.01	0.30±0.03
	3-Methyl-1-butanol-M	C123513	1213.0	Monomer	C5H12O	0.58±0.01	0.76±0.00**
	3-Methyl-1-butanol-D	C123513	1213.0	Dimer	C5H12O	1.20±0.07	1.38±0.26*
	1-Penten-3-ol	C616251	1165.9		C5H10O	0.18±0.00	0.19±0.00
	1-Butanol-M	C71363	1151.1	Monomer	C4H10O	0.48±0.01	0.70±0.04**

	1-Butanol-D	C71363	1151.8	Dimer	C4H10O	0.64±0.01	0.96±0.06**
	2-Methyl-1-propanol-M	C78831	1101.2	Monomer	C4H10O	0.09±0.01	0.15±0.03
	2-Methyl-1-propanol-D	C78831	1100.8	Dimer	C4H10O	1.08±0.01	1.27±0.06*
	1-Propanol-M	C71238	1040.4	Monomer	C3H8O	0.44±0.01	0.52±0.04
	1-Propanol-D	C71238	1041.7	Dimer	C3H8O	0.33±0.03	0.27±0.07
	2-Butanol-M	C78922	1026.1	Monomer	C4H10O	0.15±0.01	0.22±0.01**
	2-Butanol-D	C78922	1025.5	Dimer	C4H10O	0.17±0.01*	0.15±0.01
	Ethanol	C64175	943.9		C2H6O	0.76±0.20	0.42±0.05
	Total					10.21±0.25	11.74±0.28
Ketones	2-Hydroxy-3-methyl-2-cyclopenten-1-one	C80717	1923.2		C6H8O2	0.23±0.02**	0.16±0.00
	2-Undecanone	C112129	1701.8		C11H22O	0.21±0.02	0.23±0.02
	2-Heptanone	C110430	1183.6		C7H14O	0.44±0.02	0.55±0.03*
	2,3-Heptanedione	C96048	1150.4		C7H12O2	0.33±0.02	0.49±0.02**
	2-Butanone	C78933	915.2		C4H8O	1.06±0.02	1.63±0.06**
	Acetone	C67641	827.0		C3H6O	2.37±0.05	3.14±0.09**
	1-Penten-3-one	C1629589	1016.7		C5H8O	0.05±0.02	0.04±0.00
	Total					4.70±0.09	6.23±0.13**
Acids	Butanoic acid	C107926	1778.3		C4H8O2	0.75±0.05	0.74±0.05
	Acetic acid-M	C64197	1505.3	Monomer	C2H4O2	2.54±0.10	2.74±0.13
	Acetic acid-D	C64197	1505.3	Dimer	C2H4O2	1.94±0.24	1.61±0.11
	Total					5.24±0.32	5.08±0.29
Esters	Butyl acetate	C123864	1085.0		C6H12O2	0.38±0.01	0.41±0.02
	Ethyl acetate	C141786	894.1		C4H8O2	0.62±0.17*	0.13±0.02
	Methyl acetate	C79209	855.2		C3H6O2	0.28±0.02**	0.13±0.01
	Total					1.29±0.19*	0.66±0.04
Heterocycles	2,3,5-Trimethylpyrazine	C14667551	1430.5		C7H10N2	0.07±0.00	0.09±0.00**
	Pyridine	C110861	1189.3		C5H5N	0.19±0.02	0.25±0.01*
	2-Amylfuran	C3777693	1235.1		C9H14O	0.61±0.05	0.53±0.04
	Total					0.87±0.03	0.87±0.04
Alkenes	alpha-Terpinene	C99865	1191.5		C10H16	0.09±0.01	0.25±0.01**
	beta-Myrcene	C123353	1178.7		C10H16	0.06±0.01	0.05±0.01
	alpha-Phellandrene-M	C99832	1162.1	Monomer	C10H16	0.20±0.01	0.30±0.01**
	alpha-Phellandrene-D	C99832	1161.0	Dimer	C10H16	0.06±0.01	0.12±0.01**
	beta-Pinene-M	C127913	1128.6	Monomer	C10H16	0.22±0.00	0.32±0.00**
	beta-Pinene-D	C127913	1129.0	Dimer	C10H16	0.21±0.02	0.27±0.02
	Total					0.83±0.04	1.30±0.03**
Unidentifie	1		1410.8			0.19±0.01	0.22±0.01*

d	2	1352.8	0.12±0.00	0.14±0.01*
	3	1311.4	0.08±0.00	0.09±0.00*
	4	1285.8	0.14±0.01**	0.09±0.01
	5	1187.1	0.08±0.00	0.11±0.01*
	6	1181	0.13±0.01	0.12±0.00
	7	1209.8	0.24±0.01	0.29±0.04
	8	1187.2	0.20±0.03	0.27±0.05
	9	1169.6	0.24±0.01	0.30±0.01**
	10	1169.3	0.08±0.01	0.10±0.01
	11	1161	0.25±0.02	0.26±0.01
	12	1129.7	0.30±0.02	0.33±0.03
	13	1105.9	0.08±0.00	0.12±0.01**
	14	1103.8	0.55±0.04	0.55±0.09
	15	1051.8	1.76±0.03	1.50±0.16
	16	1041.7	0.21±0.01	0.26±0.01*
	17	999.1	1.78±0.04	2.07±0.05**
	18	1015.8	0.09±0.02	0.15±0.01*
	Total		6.52±0.13	6.99±0.25

Note: Values were calculated with three biological replicates; * indicates significant correlation at the level of 0.05; ** indicates significant correlation at the level of 0.01.

Table S3. Fatty acids composition during mycelial stage of *L. edodes* 808 and ww808

Compounds	Relative contents (μg/g dry weight)	
	808	ww808
Hexanoic acid (C6:0)	3.71±0.29	11.40±0.96**
Octanoic acid (C8:0)	4.21±0.82	11.66±0.24**
Decanoic acid (C10:0)	1.43±0.60	3.10±0.20*
Undecanoic acid (C11:0)	0.23±0.03	1.10±0.02**
Dodecanoic acid (C12:0)	4.85±0.40	8.56±0.37**
Tridecanoic acid (C13:0)	0.59±0.06	1.66±0.08**
Myristic acid (C14:0)	0.62±0.03	1.22±0.09**
9-Pentadecanoic acid (C15:0)	335.01±6.62	421.49±13.16**
Hexadecanoic acid (C16:0)	4316.27±178.94	4776.65±203.39*
Heptadecanoic acid (C17:0)	24.63±0.48	201.85±2.15**
Stearic acid (C18:0)	38.24±1.99	92.88±2.63**
Eicosanoic acid (C20:0)	1.66±0.03	2.96±0.04**
Heneicosanoic acid (C21:0)	0.35±0.05	0.77±0.02**
Docosanoic acid (C22:0)	3.51±0.09	5.27±0.15**
Tricosanoic acid (C23:0)	1.36±0.06	2.75±0.07**
Tetracosanoic acid (C24:0)	26.72±0.52	42.15±0.60**
Pentacosanoic acid (C25:0)	3.45±0.32	7.95±0.41**
Saturated fatty acids (SFA)	4766.86±174.06	5593.42±213.82**

Tetradecenoicacid acid (C14:1)	66.91±1.53	168.26±9.10**
(Z)-10-pentadecenoic acid (C15:1)	0.27±0.05	0.36±0.09
9-Hexadecenoic acid (C16:1)	21.78±0.80	53.82±2.49**
cis-10-Heptadecenoic acid (C17:1)	1.49±0.10	4.34±0.23**
9-Octadecenoic acid （C18:1 n9）	236.34±0.98*	212.07±7.92
11-Eicosenoic acid (C20:1）	0.79±0.10*	0.61±0.04
13-Docosenoic acid (C22:1 n9)	1.85±0.08	2.28±0.10**
15-Tetracosenoic acid (C24:1 n9)	0.73±0.06**	0.37±0.04
Monounsaturated fatty acids (MUFA)	330.17±1.88	442.12±14.99**
9,12-Octadecadienoic acid (C18:2)	842.12±26.20	917.91±21.24*
Gamma linolenic acid (C18:3 n6)	2.41±0.03**	0.14±0.02
9,12,15-Octadecatrienoic acid (C18:3 n3)	0.27±0.05	1.75±0.09**
5,8,11,14-Eicosatetraenoic acid (C20:4 n6)	5.41±0.90	9.10±0.54**
5,8,11,14,17-Eicosapentaenoic (C20:5 n3)	1.65±0.26	4.39±0.19**
Eicosatrienoic acid (C20:3)	0.01±0.00	0.03±0.00**
cis-11,14-Eicosadienoic acid (C20:2)	0.32±0.07	0.87±0.02**
4,7,10,13,16,19-Docosahexaenoic acid (C22:6n3)	5.35±0.39	7.99±0.60**
cis-13,16-Docasadienoic acid (C22:2)	0.04±0.01	0.05±0.02
Polyunsaturated fatty acids (PUFA)	857.59±26.71	942.22±20.53*
Total fatty acids (TFA)	5954.61±190.81	6977.76±241.31**

Note: Values were calculated with three biological replicates; * indicates significant correlation at the level of 0.05; ** indicates significant correlation at the level of 0.01.
